# Modern perioperative medicine – past, present, and future

**DOI:** 10.1515/iss-2019-0014

**Published:** 2019-12-05

**Authors:** Harry F. Dean, Fiona Carter, Nader K. Francis

**Affiliations:** Department of General Surgery, Yeovil District Hospital, Higher Kingston, Yeovil, UK; Enhanced Recovery after Surgery Society (UK) c.i.c., Yeovil, UK; Department of General Surgery, Yeovil District Hospital, Higher Kingston, Yeovil BA21 4AT, UK; Enhanced Recovery after Surgery Society (UK) c.i.c., Yeovil BA20 2RH, UK; School of Social and Community Medicine, University of Bristol, Canynge Hall, 39 Whatley Road, Bristol BS8 2PS, UK, Tel.: (01935) 384244

**Keywords:** enhanced recovery, ERAS, perioperative medicine, surgery

## Abstract

Modern perioperative medicine has dramatically altered the care for patients undergoing major surgery. Anaesthetic and surgical practice has been directed at mitigating the surgical stress response and reducing physiological insult. The development of standardised enhanced recovery programmes combined with minimally invasive surgical techniques has lead to reduction in length of stay, morbidity, costs, and improved outcomes. The enhanced recovery after surgery (ERAS) society and its national chapters provide a means for sharing best practice in this field and developing evidence based guidelines. Research has highlighted persisting challenges with compliance as well as ensuring the effectiveness and sustainability of ERAS. There is also a growing need for increasingly personalised care programmes as well as complex geriatric assessment of frailer patients. Continuous collection of outcome and process data combined with machine learning, offers a potentially powerful solution to delivering bespoke care pathways and optimising individual management. Long-term data from ERAS programmes remain scarce and further evaluation of functional recovery and quality of life is required.

## Introduction

The field of perioperative medicine has undergone radical change in the last 30 years witnessing major advances in anaesthetic and surgical technique. Along with this, traditional models of care have been disbanded in favour of enhanced recovery programmes in almost every surgical specialty. In this review, we have presented the evolution of modern perioperative care; we have discussed current practice, areas of contention, and future directions for advancing the field.

## Advances in perioperative care

Historically, patients knew very little of what to expect following a major surgery. Perioperative care was often characterised by prolonged fasting, aggressive bowel preparation, nasogastric decompression, bed-rest, and prolonged convalescence. A paradigm shift came during the 1990s with the work of Henrik Kehlet on the physiological stress response and organ dysfunction following surgery. He hypothesised this was a key factor in postoperative morbidity and that combined approaches to inhibit this response would improve clinical outcomes [1], [2].

Kehlet and others pioneered modern recovery – advocating multimodal analgesia and regional anaesthetic techniques [3] combined with early mobilisation and reintroduction of feeding after surgery. Utilising this approach, they reported a reduction in hospital stay for elective colectomy from 10 to 2 days [4], [5]. This heralded a new era of ‘fast track surgery’ with rapid postoperative recovery facilitated by a series of evidence based interventions delivered by a multidisciplinary team.

## Enhanced recovery after surgery

**Figure 1:**
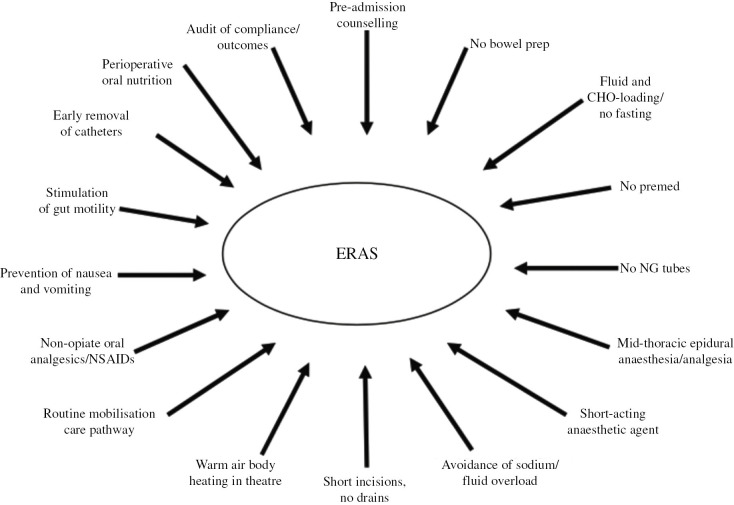
From Fearon K, Clin Nutr. 2005;24(3):466–477.

With a growing desire to reduce the morbidity and costs associated with longer hospital stay, a range of measures to optimise patient performance and recovery were investigated. In 2001 Ken Fearon and Olle Ljungqvist formed the enhanced recovery after surgery (ERAS^®^) study group. The group sought to address the variability [6] and lack of standardisation [7] in the clinical care of patients undergoing colorectal surgery. The first ERAS^®^ consensus protocol was published in 2005 [8]. It utilised multiple interventions derived from available research evidence to mitigate the perioperative physiological stress response and preserve anabolic homeostasis. These span the entire journey of a patient from preadmission to the preoperative, intraoperative, and the postoperative periods. The model is based on an integrated, multimodal approach with each of the elements combining in a synergistic and coordinated fashion, rather than acting in isolation (Figure 1). Critically, this protocol also stressed the multidisciplinary nature of perioperative care, the need for effective team structures and collaboration with stakeholders.

Alongside the rise of fast track and enhanced recovery programmes, the advent of minimally invasive and laparoscopic techniques has been a key development in gastrointestinal surgery. This has been the subject of much research [9], [10], [11] and has become the default approach in most centres. The growing use of laparoscopy-assisted surgery has been complimentary to the ERAS model [12], [13], [14] which has been reflected in the subsequent updates to the colorectal guidelines [15], [16], [17].

Despite the apparent benefits of enhanced recovery programmes, their use was initially met with scepticism and resistance [18]. Issues with implementation and poor compliance with recommendations [19] has meant ERAS is yet to fulfil its full potential in many areas. Robust population data and metaanalysis supporting ERAS in colorectal surgery have only recently become available [20], [21], [22]. As the introduction of ERAS programmes has become more structured [23] and sustainable [24], the benefits have also become increasingly evident. Data now supports an overall reduction in the length of stay across a range of surgical procedures by approximately 2.5 days without an increase in hospital readmission [20], [21], [22], [25], [26], [27], [28]. Analysis consistently demonstrates the decreased morbidity with ERAS by as much as 50%, [20], [21], [22], [25], [26], [27], [28] equivalent to one complication being avoided for every 4.5 patients following an enhanced recovery programme [25]. This has also been associated with significant cost cutting, either through the direct reduction in complications or through more efficient utilisation of resources and availability of hospital beds [25], [28], [29], [30].

## Barriers to success

Following the foundation of the ERAS society in 2010, experts in the fields of hepatobillary surgery [31], [32], gynaecology [33], urology [34], head and neck [35], breast reconstruction [36] and cardiothoracic surgery [37], [38] have authored guidelines and ERAS protocols, which are successfully in place in more than 25 countries worldwide. Whilst this may be regarded as a success, data collected from this expansion has revealed perhaps unsurprisingly, that protocols which are incompletely delivered or inchoate are markedly reduced in their effectiveness [19], [39], [40].

To combat this, a renewed emphasis has been placed on quality assurance with greater scrutiny in the implementation process, adherence and sustainability of ERAS. Successful implementation requires the training and education of staff members as well as effective change management strategies and good clinical leadership [19], [41], [42]. A number of training resources have been developed to accomplish this including the ERAS implementation programme and interactive audit system and more recently, a detailed consensus framework for the optimal training curriculum [43].

Greater compliance with ERAS programmes is directly associated with decreased complication rates and improved outcomes in a ‘dose dependent’ manner [39], [44], [45]. Repeated audit and feedback of centre-specific outcome and process data is therefore a vital means of ensuring the quality and sustainability of ERAS. The ERAS interactive audit system was developed to help centres monitor adherence to ERAS, to enable benchmarking between institutions and to confirm the legitimacy of ERAS out of the trial setting. The system holds an international database, which has become a valuable tool for research as well as directing the improvement and development of ERAS [46], [47].

## National societies

The ERAS Society (UK) was formed in 2009, following a national initiative (Enhanced Recovery Partnership Programme, ERPP) [48] for the spread and adoption of ERAS principles for key procedures across four specialties (colorectal, gynaecology, urology, orthopaedics). A Delphi study with healthcare staff involved in the ERPP showed consensus for a continued means for future networking and information sharing [49]. ERAS UK have run annual conferences since 2010, with each event held in different regions of the UK, involving local healthcare professionals and have seen an expanding membership across all surgical specialties. Networking opportunities at these national conferences result in new research collaborations [50] and enable groups to share pathways, protocols, and documentation.

ERAS UK have also explored other ways to enable information sharing with online resources available on their website (www.erasuk.net), a closed Facebook group for discussion (www.facebook.com/groups/erasuk) and a more secure forum with a searchable document library (www.khub.net/group/enhanced-recovery-after-surgery-society-uk). Direct interaction with ERAS UK members, plus social media discussions on Facebook and Twitter (@ERASsocietyUK) ensure that the society continues to evolve and spread awareness of this multimodal model of care.

## Disparity and debate

There are several areas of continuing debate in perioperative care, for example the usage of mechanical bowel preparation (MBP). European ERAS guidelines do not currently recommend routine use of bowel preparation for colonic resection [16], [51]. However, recent systematic reviews suggest that MBP combined with oral antibiotic therapy may be effective in reducing surgical site infection and other postoperative complications [52], [53], [54]. This analysis has been well received by American consensus groups who advocate this as the preferred preparation for elective colonic surgery [55].

Other areas of controversy include intraoperative fluid therapy, which has been an important component of ERAS from its inception. Balancing fluid therapy to achieve adequate splanchnic perfusion whilst avoiding oedema, paralytic ileus, and fluid overload remains challenging. This is particularly true amongst high-risk patients such as those with limited physiologic reserve, severe cardiopulmonary disease, renal impairment, or patients undergoing extensive surgery [56]. Early trials suggested that goal directed fluid therapy (GDFT), which makes use of advanced monitoring systems and fluid boluses to achieve a targeted cardiac output, may confer an advantage over traditional care. However, contemporary studies comparing GDFT to enhanced recovery patients receiving evidence-based fluid management in the form of preoperative euvolaemia and neutral fluid balance have shown no significant difference [56], [57], [58]. The relative equipoise in the literature has again lead to geographic disparity with American societies recommending GDFT as standard [55], [59] and the European ERAS community reserving GDFT for high risk patients only [17].

## Evolution

ERAS challenged all the dogmas of conventional perioperative care, but it needs to continue to evolve, or else risk becoming dogmatic itself. ERAS must be responsive to the latest research evidence as well as novel surgical approaches and technologies. Enhanced recovery programmes have already begun to influence the care of emergency general surgery [60], [61] and paediatric patients [62]. The use of robotic surgery [63], [64], trans anal [65], and other minimally invasive techniques is likely to alter the surgical insult and patient recovery as surgical expertise with these procedures grow.

Research within ERAS is also evolving with on-going work to investigate ways of enhancing the success of ERAS. Studies to predict which patients are likely to deviate from the expected perioperative course has been of interest as it may allow remedial action to be taken to avert this [66]. Recent studies suggest that compliance is worst in the immediate postoperative period following colorectal surgery and may be most indicative of early complication or impaired functional recovery [67], [68]. Whilst this is a potential area for significant improvement, it remains unclear whether ‘non-compliance’ in these instances was due to a perioperative complication or whether a complication resulted from poor compliance [69]. More structured reporting of outcomes is likely to assist with this enquiry as well as answering research questions into the benefit of specific ERAS components [70].

Deconstructing ERAS protocols by analysing the effectiveness of specific items may seem counterintuitive given the evidence base is required to justify each component. Efforts to simplify and streamline protocols are primarily borne from a desire to improve compliance, but may also represent a response to criticism that ERAS has become overcomplicated and unwieldy [71]. Debate has also arisen as to whether early tolerance of oral intake and early mobilisation should be considered as markers of adherence or as markers of recovery [69].

## Personalised perioperative care

What is increasingly clear in perioperative medicine is that one size does not fit all. The individual stress response to surgery remains highly variable without a means to measure or predict this currently. The increasing complexity of patients’ medical needs combined with heterogeneity in service infrastructure, operative, and patient factors are driving a need for more personalised care programs. This is particularly true in the context of an aging global population and the rising number of elderly and comorbid patients undergoing surgery. The increasing prevalence of frailty and geriatric syndromes amongst this patient group places them at increased risk of adverse outcome following surgery including medical complications, prolonged hospitalisation, institutionalisation, and readmission as well as short and long-term mortality [72].

Although series have demonstrated that ERAS is safe and beneficial in caring for elderly surgical patients [73], a number of studies support the use of multidomain comprehensive geriatric assessment (CGA) to identify and manage older patients who are at the risk of elective surgery [74], [75], [76]. Several centres have successfully combined this with embedded liaison services to deliver geriatrician lead, evidence based, collaborative models of care throughout the perioperative period [77], [78]. These teams contribute to preoperative assessment and medical optimisation, counselling and shared decision making, inpatient review and rehabilitation as well as proactive discharge planning. The role of perioperative geriatricians has become well established in patients undergoing emergency surgery for hip fracture, but is likely to extend to frail and elderly patients undergoing major surgery in vascular and general surgery as well. Jugdeep Dhesi and others have demonstrated significant reductions in complication rates, length of stay, and likelihood of discharge to dependent care settings with CGA methods [75], [77], [78]. In order to meet the needs of these patients personalised, holistic care programs are required necessitating closer collaboration between perioperative geriatricians, anaesthetists, and surgeons.

## Future directions in perioperative care

The growing complexity of perioperative care and the need for increasingly personalised and bespoke pathways has stimulated interest in digital technological solutions and automated care processes. Digital technology is likely to have a major role in shaping the future of perioperative care and a number of advances relevant to enhanced recovery programmes have been investigated. These include apps directed at lifestyle modification and preoperative optimisation, objective nociceptive measurements, portable non-invasive sensors calibrated to recognise postoperative cardiopulmonary complications, and activity trackers to monitor postoperative ambulatory recovery [79].

An exciting frontier in medical technology is in combining big data analytics with artificial intelligence in order to guide patient management. Artificial neural networks and machine learning programmes have exhibited superior performance to conventional prediction models in diagnosing acute appendicitis [80], selecting patients for surgery [81], predicting quality of life after breast cancer surgery [82] and long term mortality following surgery for hepatocellular carcinoma [83]. A centre in New Jersey, USA implemented a machine-learning algorithm in the emergency department, intensive care unit, and hospital wards to identify patients with sepsis earlier. They were able to reduce the sepsis-related in-hospital mortality rate by 60% and sepsis-related 30-day readmission rate by 50% [84]. Similar models have also been applied retrospectively to colorectal ERAS patients using multi-layered perception neural networks to calculate the individual prehabilitation windows as well as the probability of delayed discharge and readmission [85], [86].

**Figure 2:**
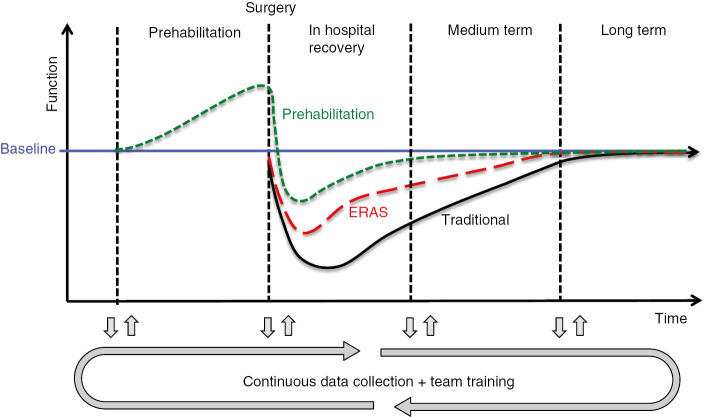
Model of the effect of prehabilitation and ERAS on functional recovery.

This may well revolutionise care of the surgical patient within the next 20 years. We envisage a system whereby data from electronic health records combined with metrics prospectively measured throughout the perioperative period are harvested by machine-learning programmes. This continuously updates the optimum care pathway with targeted adaptations or adjustments for an individual patient as well as the local population in an automated fashion (Figure 2).

There is cause for great optimism about the future of perioperative medicine. There has been greater consensus and collaboration on issues such as nutrition [87], anaemia [88], as well as procedure-specific pain management [89]. Prehabilitation – which is reviewed by Gerrit Slooter in this edition of the journal, is a particularly promising means of reducing perioperative morbidity. It is worth highlighting however, whilst enhanced recovery programmes have dramatically altered surgical practice, this has been a relatively recent change and there is a distinct lack of data regarding their long-term outcomes [90]. ERAS research to date has almost exclusively focused on length of stay, readmission rate and 30-day morbidity and mortality. However, these measures fail to reflect the complex, multidimensional process of recovery after surgery. To better understand and influence the recovery continuum more detailed assessment of physical, nociceptive, emotive, functional and cognitive performance at multiple time points are required [91].

Studies examining patient satisfaction and health related quality of life with ERAS have so far found no significant difference when compared to conventional care. Some evidence supports a reduction in postoperative fatigue [92] and earlier return to activities with ERAS however, a small number of studies have reported slightly higher pain scores with ERAS in the early postoperative period [93]. Although this difference disappeared with time, this is clearly an area for potential improvement as dissatisfaction associated with postoperative pain can persist long after the index surgery [94].

A limited number of reports have now been published examining medium to long-term survival, which reveals both an overall and cancer-specific survival advantage with ERAS [95], [96], [97]. It is likely that this effect is due to direct reduction in complications and in preventing delay to commencing adjuvant chemotherapy. This finding may also result from minimising the surgical stress response placed on the immune system and the complex ways in which this modulates tumour biology.

Crucially these studies have reasserted the importance of adherence to ERAS protocols which was strongly associated with survival independent of cancer stage and postoperative complications [45], [96], [97].

From a global perspective there is still considerable progress required in perioperative care. In 2015 the Lancet commission on global surgery highlighted the alarming deficit in essential, life-saving surgical and anaesthesia care in low and middle income countries [98]. The report estimated that 5 billion people lack access to safe, affordable surgical, and anaesthesia care when needed. Achieving the commissions aims has been aided by commitments from the world health organisation [99] and most recently from the world bank group [100]. Application of ERAS principles may also have a role in supporting the optimal growth and development of surgical systems as they are scaled-up within National Surgery Plans [101]. However, a broad interdisciplinary focus is still urgently required to address the health system requirements for patients with surgical conditions worldwide [102].

## Supplementary Material

The article (https://doi.org/10.1515/iss-2019-0014) offers reviewer assessments as supplementary material.

## References

[1] Kehlet H The stress response to surgery: release mechanisms and the modifying effect of pain relief. Acta Chir Scand Suppl 1989;550:22–8. 2652970

[2] Kehlet H Multimodal approach to control postoperative pathophysiology and rehabilitation. Br J Anaesth 1997;78:606–17. 9175983 10.1093/bja/78.5.606

[3] Kehlet H , Dahl JB The value of “multimodal” or “balanced analgesia” in postoperative pain treatment. Anesth Analg 1993;77:1048–56. 8105724 10.1213/00000539-199311000-00030

[4] Bardram L , Funch-Jensen P , Jensen P , Kehlet H , Crawford M Recovery after laparoscopic colonic surgery with epidural analgesia, and early oral nutrition and mobilisation. Lancet 1995;345:763–4. 7891489 10.1016/s0140-6736(95)90643-6

[5] Kehlet H , Mogensen T Hospital stay of 2 days after open sigmoidectomy with a multimodal rehabilitation programme. Br J Surg 1999;86:227–30. 10100792 10.1046/j.1365-2168.1999.01023.x

[6] Lassen K , Hannemann P , Ljungqvist O , Fearon K , Dejong CH , von Meyenfeldt MF , Patterns in current perioperative practice: survey of colorectal surgeons in five northern European countries. Br Med J 2005;330:1420–1. 15911535 10.1136/bmj.38478.568067.AE PMC558375

[7] Nygren J , Hausel J , Kehlet H , Revhaug A , Lassen K , Dejong C , A comparison in five European Centres of case mix, clinical management and outcomes following either conventional or fast-track perioperative care in colorectal surgery. Clin Nutr 2005;24:455–61. 15896433 10.1016/j.clnu.2005.02.003

[8] Fearon KC , Ljungqvist O , Von Meyenfeldt M , Revhaug A , Dejong CH , Lassen K , Enhanced recovery after surgery: a consensus review of clinical care for patients undergoing colonic resection. Clin Nutr 2005;24:466–77. 15896435 10.1016/j.clnu.2005.02.002

[9] Kuhry E , Schwenk W , Gaupset R , Romild U , Bonjer J Long-term outcome of laparoscopic surgery for colorectal cancer: a cochrane systematic review of randomised controlled trials. Cancer Treat Rev 2008;34:498–504. 18468803 10.1016/j.ctrv.2008.03.011

[10] Colon Cancer Laparoscopic or Open Resection Study Group , Buunen M , Veldkamp R , Hop WC , Kuhry E , Jeekel J , Survival after laparoscopic surgery versus open surgery for colon cancer: long-term outcome of a randomised clinical trial. Lancet Oncol 2009;10:44–52. 19071061 10.1016/S1470-2045(08)70310-3

[11] Green BL , Marshall HC , Collinson F , Quirke P , Guillou P , Jayne DG , Long-term follow-up of the Medical Research Council CLASICC trial of conventional versus laparoscopically assisted resection in colorectal cancer. Br J Surg 2013;100:75–82. 23132548 10.1002/bjs.8945

[12] King PM , Blazeby JM , Ewings P , Franks PJ , Longman RJ , Kendrick AH , Randomized clinical trial comparing laparoscopic and open surgery for colorectal cancer within an enhanced recovery programme. Br J Surg 2006;93:300–8. 16363014 10.1002/bjs.5216

[13] Buchanan GN , Malik A , Parvaiz A , Sheffield JP , Kennedy RH Laparoscopic resection for colorectal cancer. Br J Surg 2008;95:893–902. 18551725 10.1002/bjs.6019

[14] Vlug M , Wind J , Hollmann M DU-A of, 2011 undefined. Laparoscopy in combination with fast track multimodal management is the best perioperative strategy in patients undergoing colonic surgery: a randomized clinical, journals.lww.com. https://journals.lww.com/annalsofsurgery/FullText/2011/12000/Laparoscopy_in_Combination_with_Fast_Track.7.aspx Accessed July 4, 2019. 10.1097/SLA.0b013e31821fd1ce 21597360

[15] Lassen K , Soop M , Nygren J , Consensus review of optimal perioperative care in colorectal surgery enhanced recovery after surgery (ERAS) group recommendations. Arch Surg 2009;144:961. 19841366 10.1001/archsurg.2009.170

[16] Gustafsson UO , Scott MJ , Schwenk W , Demartines N , Roulin D , Francis N , Guidelines for perioperative care in elective colonic surgery: enhanced recovery after surgery (ERAS^®^) society recommendations. Clin Nutr 2012;31:783–800. 23099039 10.1016/j.clnu.2012.08.013

[17] Gustafsson UO , Scott MJ , Hubner M , Nygren J , Demartines N , Francis N , Guidelines for perioperative care in elective colorectal surgery: enhanced recovery after surgery (ERAS^®^) society recommendations: 2018. World J Surg 2019;43:659–95. 30426190 10.1007/s00268-018-4844-y

[18] Pearsall EA , Meghji Z , Pitzul KB , Aarts MA , McKenzie M , McLeod RS , A qualitative study to understand the barriers and enablers in implementing an enhanced recovery after surgery program. Ann Surg 2015;261:92–6. 24646564 10.1097/SLA.0000000000000604

[19] Maessen J , Dejong CH , Hausel J , Nygren J , Lassen K , Andersen J , A protocol is not enough to implement an enhanced recovery programme for colorectal resection. Br J Surg 2007;94:224–31. 17205493 10.1002/bjs.5468

[20] Varadhan KK , Neal KR , Dejong CH , Fearon KC , Ljungqvist O , Lobo DN The enhanced recovery after surgery (ERAS) pathway for patients undergoing major elective open colorectal surgery: a meta-analysis of randomized controlled trials. Clin Nutr 2010;29:434–40. 20116145 10.1016/j.clnu.2010.01.004

[21] Spanjersberg WR , Reurings J , Keus F , van Laarhoven CJ Fast track surgery versus conventional recovery strategies for colorectal surgery. Cochrane Database Syst Rev 2011;2:CD007635. 10.1002/14651858.CD007635.pub2 PMC13061361 21328298

[22] Greco M , Capretti G , Beretta L , Gemma M , Pecorelli N , Braga M Enhanced recovery program in colorectal surgery: a meta-analysis of randomized controlled trials. World J Surg 2014;38:1531–41. 24368573 10.1007/s00268-013-2416-8

[23] Gillissen F , Hoff C , Maessen JM , Winkens B , Teeuwen JH , von Meyenfeldt MF , Structured synchronous implementation of an enhanced recovery program in elective colonic surgery in 33 hospitals in The Netherlands. World J Surg 2013;37:1082–93. 23392451 10.1007/s00268-013-1938-4

[24] Gillissen F , Ament SM , Maessen JM , Dejong CH , Dirksen CD , van der Weijden T , Sustainability of an enhanced recovery after surgery program (ERAS) in colonic surgery. World J Surg 2015;39:526–33. 25148885 10.1007/s00268-014-2744-3

[25] Adamina M , Kehlet H , Tomlinson GA , Senagore AJ , Delaney CP Enhanced recovery pathways optimize health outcomes and resource utilization: a meta-analysis of randomized controlled trials in colorectal surgery. Surgery 2011;149:830–40. 21236454 10.1016/j.surg.2010.11.003

[26] Nicholson A , Lowe MC , Parker J , Lewis SR , Alderson P , Smith AF Systematic review and meta-analysis of enhanced recovery programmes in surgical patients. Br J Surg 2014;101:172–88. 24469618 10.1002/bjs.9394

[27] Li Z , Zhao Q , Bai B , Ji G , Liu Y Enhanced recovery after surgery programs for laparoscopic abdominal surgery: a systematic review and meta-analysis. World J Surg 2018;42:3463–73. 29750324 10.1007/s00268-018-4656-0

[28] Visioni A , Shah R , Gabriel E , Attwood K , Kukar M , Nurkin S Enhanced recovery after surgery for noncolorectal surgery? A systematic review and meta-analysis of major abdominal surgery. Ann Surg 2018;267:57–65. 28437313 10.1097/SLA.0000000000002267

[29] Stephen AE , Berger DL Shortened length of stay and hospital cost reduction with implementation of an accelerated clinical care pathway after elective colon resection. Surgery 2003;133:277–82. 12660639 10.1067/msy.2003.19

[30] Thanh NX , Chuck AW , Wasylak T , Lawrence J , Faris P , Ljungqvist O , An economic evaluation of the enhanced recovery after surgery (ERAS) multisite implementation program for colorectal surgery in Alberta. Can J Surg 2016;59:415–21. 28445024 10.1503/cjs.006716 PMC5125924

[31] Lassen K , Coolsen MM , Slim K , Carli F , de Aguilar-Nascimento JE , Schäfer M , Guidelines for perioperative care for pancreaticoduodenectomy: Enhanced recovery after surgery (ERAS^®^) society recommendations. Clin Nutr 2012;31:817–30. 23079762 10.1016/j.clnu.2012.08.011

[32] Melloul E , Hübner M , Scott M , Snowden C , Prentis J , Dejong CH , Guidelines for perioperative care for liver surgery: enhanced recovery after surgery (ERAS) society recommendations. World J Surg 2016;40:2425–40. 27549599 10.1007/s00268-016-3700-1

[33] Nelson G , Altman AD , Nick A , Meyer LA , Ramirez PT , Achtari C , Guidelines for pre- and intra-operative care in gynecologic/oncology surgery: enhanced recovery after surgery (ERAS^®^) society recommendations – Part I. Gynecol Oncol 2016;140:313–22. 26603969 10.1016/j.ygyno.2015.11.015

[34] Cerantola Y , Valerio M , Persson B , Jichlinski P , Ljungqvist O , Hubner M , Guidelines for perioperative care after radical cystectomy for bladder cancer: enhanced recovery after surgery (ERAS^®^) society recommendations. Clin Nutr 2013;32:879–87. 24189391 10.1016/j.clnu.2013.09.014

[35] Dort JC , Farwell DG , Findlay M , Huber GF , Kerr P , Shea-Budgell MA , Optimal perioperative care in major head and neck cancer surgery with free flap reconstruction. JAMA Otolaryngol Neck Surg 2017;143:292. 10.1001/jamaoto.2016.2981 27737447

[36] Weiser MR , Gonen M , Usiak S , Pottinger T , Samedy P , Patel D , Effectiveness of a multidisciplinary patient care bundle for reducing surgical-site infections. Br J Surg 2018;105:1680–7. 29974946 10.1002/bjs.10896 PMC6190910

[37] Batchelor TJ , Rasburn NJ , Abdelnour-Berchtold E , Brunelli A , Cerfolio RJ , Gonzalez M , Guidelines for enhanced recovery after lung surgery: recommendations of the enhanced recovery after surgery (ERAS^®^) society and the European society of thoracic surgeons (ESTS). Eur J Cardio-thoracic Surg 2019;55:91–115. 10.1093/ejcts/ezy301 30304509

[38] Engelman DT , Ben Ali W , Williams JB , Perrault LP , Reddy VS , Arora RC , Guidelines for perioperative care in cardiac surgery: enhanced recovery after surgery society recommendations. JAMA Surg 2019;154:755–66. 31054241 10.1001/jamasurg.2019.1153

[39] Gustafsson UO , Hausel J , Thorell A , Ljungqvist O , Soop M , Nygren J , Adherence to the enhanced recovery after surgery protocol and outcomes after colorectal cancer surgery. Arch Surg 2011;146:571. 21242424 10.1001/archsurg.2010.309

[40] ERAS Compliance Group. The impact of enhanced recovery protocol compliance on elective colorectal cancer resection. Ann Surg 2015;261:1153–9. 25671587 10.1097/SLA.0000000000001029

[41] Cohen R , Gooberman-Hill R Staff experiences of enhanced recovery after surgery: systematic review of qualitative studies. BMJ Open 2019;9:e022259. 10.1136/bmjopen-2018-022259 PMC6377558 30760511

[42] Gotlib Conn L , McKenzie M , Pearsall EA , McLeod RS Successful implementation of an enhanced recovery after surgery programme for elective colorectal surgery: a process evaluation of champions’ experiences. Implement Sci 2015;10:99. 26183086 10.1186/s13012-015-0289-y PMC4504167

[43] Francis NK , Walker T , Carter F , Hübner M , Balfour A , Jakobsen DH , Consensus on training and implementation of enhanced recovery after surgery: a Delphi study. World J Surg 2018;42:1919–28. 29302724 10.1007/s00268-017-4436-2

[44] Pisarska M , Pędziwiatr M , Małczak P , Major P , Ochenduszko S , Zub-Pokrowiecka A , Do we really need the full compliance with ERAS protocol in laparoscopic colorectal surgery? A prospective cohort study. Int J Surg 2016;36:377–82. 27876677 10.1016/j.ijsu.2016.11.088

[45] Ripollés-Melchor J , Ramírez-Rodríguez JM , Casans-Francés R , Aldecoa C , Abad-Motos A , Logroño-Egea M , Association between use of enhanced recovery after surgery protocol and postoperative complications in colorectal surgery. JAMA Surg 2019. 10.1001/jamasurg.2019.0995 PMC6506896 31066889

[46] Currie A , Burch J , Jenkins JT , Faiz O , Kennedy RH , Ljungqvist O , The impact of enhanced recovery protocol compliance on elective colorectal cancer resection: results from an international registry. Ann Surg 2015;261:1153–9. 25671587 10.1097/SLA.0000000000001029

[47] Currie A , Soop M , Demartines N , Fearon K , Kennedy R , Ljungqvist O Enhanced recovery after surgery interactive audit system: 10 years’ experience with an international web-based clinical and research perioperative care database. Clin Colon Rectal Surg 2019;32:75–91. 30647549 10.1055/s-0038-1673357 PMC6327717

[48] McNaney N Enhanced recovery partnership programme 2011. http://www.dh.gov.uk/publications Accessed August 14, 2019.

[49] Knott A , Pathak S , McGrath JS , Kennedy R , Horgan A , Mythen M , Consensus views on implementation and measurement of enhanced recovery after surgery in England: Delphi study. BMJ Open 2012;2:e001878. 10.1136/bmjopen-2012-001878 PMC3533042 23242242

[50] Burch J , Fecher-Jones I , Balfour A , Fitt I , Carter F What is an enhanced recovery nurse: a literature review and audit. Gastrointest Nurs 2017;15:43–50.

[51] Nygren J , Thacker J , Carli F , Fearon KC , Norderval S , Lobo DN , Guidelines for perioperative care in elective rectal/pelvic surgery: enhanced recovery after surgery (ERAS^®^) society recommendations. World J Surg 2013;37:285–305. 23052796 10.1007/s00268-012-1787-6

[52] Chen M , Song X , Chen L , Lin Z , Zhang X Comparing mechanical bowel preparation with both oral and systemic antibiotics versus mechanical bowel preparation and systemic antibiotics alone for the prevention of surgical site infection after elective colorectal surgery. Dis Colon Rectum 2016;59:70–8. 26651115 10.1097/DCR.0000000000000524

[53] Toh JW , Phan K , Hitos K , Pathma-Nathan N , El-Khoury T , Richardson AJ , Association of mechanical bowel preparation and oral antibiotics before elective colorectal surgery with surgical site infection. JAMA Netw Open 2018;1:e183226. 30646234 10.1001/jamanetworkopen.2018.3226 PMC6324461

[54] McSorley ST , Steele CW , McMahon AJ Meta-analysis of oral antibiotics, in combination with preoperative intravenous antibiotics and mechanical bowel preparation the day before surgery, compared with intravenous antibiotics and mechanical bowel preparation alone to reduce surgical-site infec. BJS Open 2018;2:185–94. 30079387 10.1002/bjs5.68 PMC6069350

[55] Carmichael JC , Keller DS , Baldini G , Bordeianou L , Weiss E , Lee L , Clinical practice guidelines for enhanced recovery after colon and rectal surgery from the american society of colon and rectal surgeons and society of american gastrointestinal and endoscopic surgeons. Dis Colon Rectum 2017;60:761–84. 28682962 10.1097/DCR.0000000000000883

[56] Pearse RM , Harrison DA , MacDonald N , Gillies MA , Blunt M , Ackland G , Effect of a perioperative, cardiac output–guided hemodynamic therapy algorithm on outcomes following major gastrointestinal surgery. J Am Med Assoc 2014;311:2181. 10.1001/jama.2014.5305 24842135

[57] Brandstrup B , Svendsen PE , Rasmussen M , Belhage B , Rodt SÅ , Hansen B , Which goal for fluid therapy during colorectal surgery is followed by the best outcome: near-maximal stroke volume or zero fluid balance?. Br J Anaesth 2012;109:191–9. 22710266 10.1093/bja/aes163

[58] Rollins KE , Lobo DN Intraoperative goal-directed fluid therapy in elective major abdominal surgery: a meta-analysis of randomized controlled trials. Ann Surg 2016;263:465–76. 26445470 10.1097/SLA.0000000000001366 PMC4741406

[59] Thiele RH , Raghunathan K , Brudney CS , Lobo DN , Martin D , Senagore A , American society for enhanced recovery (ASER) and perioperative quality initiative (POQI) joint consensus statement on perioperative fluid management within an enhanced recovery pathway for colorectal surgery. Perioper Med 2016;5:24. 10.1186/s13741-016-0049-9 PMC5027098 27660701

[60] Roulin D , Blanc C , Muradbegovic M , Hahnloser D , Demartines N , Hübner M Enhanced recovery pathway for urgent colectomy. World J Surg 2014;38:2153–9. 24668455 10.1007/s00268-014-2518-y

[61] Quiney N , Aggarwal G , Scott M , Dickinson M Survival after emergency general surgery: What can we learn from enhanced recovery programmes?. World J Surg 2016;40:1283–7. 26813539 10.1007/s00268-016-3418-0

[62] Short HL , Heiss KF , Burch K , Travers C , Edney J , Venable C , Implementation of an enhanced recovery protocol in pediatric colorectal surgery. J Pediatr Surg 2018;53:688–92. 28545764 10.1016/j.jpedsurg.2017.05.004

[63] Polat F , Willems LH , Dogan K , Rosman C The oncological and surgical safety of robot-assisted surgery in colorectal cancer: outcomes of a longitudinal prospective cohort study. Surg Endosc 2019;33:3644–55. 30693389 10.1007/s00464-018-06653-2 PMC6795614

[64] Perez RE , Schwaitzberg SD Robotic surgery: finding value in 2019 and beyond. Ann Laparosc Endosc Surg 2019;4:51.

[65] Deijen CL , Velthuis S , Tsai A , Mavroveli S , de Lange-de Klerk ES , Sietses C , COLOR III: a multicentre randomised clinical trial comparing transanal TME versus laparoscopic TME for mid and low rectal cancer. Surg Endosc 2016;30:3210–5. 26537907 10.1007/s00464-015-4615-x PMC4956704

[66] Messenger DE , Curtis NJ , Jones A , Jones EL , Smart NJ , Francis NK Factors predicting outcome from enhanced recovery programmes in laparoscopic colorectal surgery: a systematic review. Surg Endosc 2017;31:2050–71. 27631314 10.1007/s00464-016-5205-2

[67] Roulin D , Muradbegovic M , Addor V , Blanc C , Demartines N , Hübner M Enhanced recovery after elective colorectal surgery – reasons for non-compliance with the protocol. Dig Surg 2017;34:220–6. 27941313 10.1159/000450685

[68] Aarts M-A , Rotstein OD , Pearsall EA , Victor JC , Okrainec A , McKenzie M , Postoperative ERAS interventions have the greatest impact on optimal recovery. Ann Surg 2018;267:992–7. 29303803 10.1097/SLA.0000000000002632

[69] Slim K , Joris J The egg-and-chicken situation in postoperative enhanced recovery programmes. Br J Anaesth 2017;118:5–6. 28039235 10.1093/bja/aew408

[70] Elias KM , Stone AB , McGinigle K , Tankou JI , Scott MJ , Fawcett WJ , The Reporting on ERAS compliance, outcomes, and elements research (RECOvER) checklist: a joint statement by the ERAS^®^ and ERAS^®^ USA societies. World J Surg 2019;43:1–8. 30116862 10.1007/s00268-018-4753-0 PMC6313353

[71] Kehlet H ERAS implementation – time to move forward. Ann Surg 2018;267:998–9. 29462010 10.1097/SLA.0000000000002720

[72] Chan SP , Ip KY , Irwin MG Peri-operative optimisation of elderly and frail patients: a narrative review. Anaesthesia 2019;74:80–9. 30604415 10.1111/anae.14512

[73] Ljungqvist O , Hubner M Enhanced recovery after surgery – ERAS – principles, practice and feasibility in the elderly. Aging Clin Exp Res 2018;30:249–52. 29453605 10.1007/s40520-018-0905-1 PMC5856872

[74] Partridge JS , Harari D , Martin FC , Dhesi JK The impact of pre-operative comprehensive geriatric assessment on postoperative outcomes in older patients undergoing scheduled surgery: a systematic review. Anaesthesia 2014;69:8–16. 24303856 10.1111/anae.12494

[75] Partridge JSL , Harari D , Martin FC , Peacock JL , Bell R , Mohammed A , Randomized clinical trial of comprehensive geriatric assessment and optimization in vascular surgery. Br J Surg 2017;104:679–87. 28198997 10.1002/bjs.10459

[76] Watt J , Tricco AC , Talbot-Hamon C , Pham B , Rios P , Grudniewicz A , Identifying older adults at risk of harm following elective surgery: a systematic review and meta-analysis. BMC Med 2018;16:2. 29325567 10.1186/s12916-017-0986-2 PMC5765656

[77] Harari D , Hopper A , Dhesi J , Babic-Illman G , Lockwood L , Martin F Proactive care of older people undergoing surgery (‘POPS’): designing, embedding, evaluating and funding a comprehensive geriatric assessment service for older elective surgical patients. Age Ageing 2007;36:190–6. 17259638 10.1093/ageing/afl163

[78] Shipway D , Koizia L , Winterkorn N , Fertleman M , Ziprin P , Moorthy K Embedded geriatric surgical liaison is associated with reduced inpatient length of stay in older patients admitted for gastrointestinal surgery. Future Healthc J 2018;5:108–16. 31098544 10.7861/futurehosp.5-2-108 PMC6502563

[79] Michard F , Gan TJ , Kehlet H Digital innovations and emerging technologies for enhanced recovery programmes. Br J Anaesth 2017;119:31–9. 28605474 10.1093/bja/aex140

[80] Prabhudesai SG , Gould S , Rekhraj S , Tekkis PP , Glazer G , Ziprin P Artificial neural networks: useful aid in diagnosing acute appendicitis. World J Surg 2008;32:305–9. 18043966 10.1007/s00268-007-9298-6

[81] Yoo TK , Ryu IH , Lee G , Kim Y , Kim JK , Lee IS , Adopting machine learning to automatically identify candidate patients for corneal refractive surgery. npj Digit Med 2019;2:59. 31304405 10.1038/s41746-019-0135-8 PMC6586803

[82] Tsai J-T , Hou M-F , Chen Y-M , Wan TTH , Kao H-Y , Shi H-Y Predicting quality of life after breast cancer surgery using ANN-based models: performance comparison with MR. Support Care Cancer 2013;21:1341–50. 23203653 10.1007/s00520-012-1672-8

[83] Shi H-Y , Lee K-T , Wang J-J , Sun D-P , Lee H-H , Chiu C-C Artificial neural network model for predicting 5-year mortality after surgery for hepatocellular carcinoma: a nationwide study. J Gastrointest Surg 2012;16:2126–31. 22878787 10.1007/s11605-012-1986-3

[84] McCoy A , Das R Reducing patient mortality, length of stay and readmissions through machine learning-based sepsis prediction in the emergency department, intensive care unit and hospital floor units. BMJ Open Qual 2017;6:e000158. 10.1136/bmjoq-2017-000158 PMC5699136 29450295

[85] Curtis NJ , Dennison G , Salib E , Hashimoto DA , Francis NK Artificial neural network individualised prediction of time to colorectal cancer surgery. Gastroenterol Res Pract 2019;2019:1–10. 10.1155/2019/1285931 PMC6652036 31360163

[86] Francis NK , Luther A , Salib E , Allanby L , Messenger D , Allison AS , The use of artificial neural networks to predict delayed discharge and readmission in enhanced recovery following laparoscopic colorectal cancer surgery. Tech Coloproctol 2015;19:419–28. 26084884 10.1007/s10151-015-1319-0

[87] Sandrucci S , Beets G , Braga M , Dejong K , Demartines N Perioperative nutrition and enhanced recovery after surgery in gastrointestinal cancer patients. A position paper by the ESSO task force in collaboration with the ERAS society (ERAS coalition). Eur J Surg Oncol 2018;44:509–14. 29398322 10.1016/j.ejso.2017.12.010

[88] Muñoz M , Acheson AG , Auerbach M , Besser M , Habler O , Kehlet H , International consensus statement on the peri-operative management of anaemia and iron deficiency. Anaesthesia 2017;72:233–47. 27996086 10.1111/anae.13773

[89] Lee B , Schug SA , Joshi GP , Kehlet H , PROSPECT Working Group. Procedure-specific pain management (PROSPECT) – an update. Best Pract Res Clin Anaesthesiol 2018;32:101–11. 30322452 10.1016/j.bpa.2018.06.012

[90] Neville A , Lee L , Antonescu I , Mayo NE , Vassiliou MC , Fried GM , Systematic review of outcomes used to evaluate enhanced recovery after surgery. Br J Surg 2014;101:159–71. 24469616 10.1002/bjs.9324

[91] Bowyer AJ , Royse CF Postoperative recovery and outcomes – what are we measuring and for whom?. Anaesthesia 2016;71:72–7. 26620150 10.1111/anae.13312

[92] Zargar-Shoshtari K , Paddison JS , Booth RJ , Hill AG A prospective study on the influence of a fast-track program on postoperative fatigue and functional recovery after major colonic surgery. J Surg Res 2009;154:330–5. 19118844 10.1016/j.jss.2008.06.023

[93] Li D , Jensen C Patient satisfaction and quality of life with enhanced recovery protocols. Clin Colon Rectal Surg 2019;32:138–44. 30833864 10.1055/s-0038-1676480 PMC6395092

[94] Deiss T , Chen L , Sarin A , Naidu RK Patient-reported outcomes 6 months after enhanced recovery after colorectal surgery. Perioper Med 2018;7:19. 10.1186/s13741-018-0099-2 PMC6106896 30159140

[95] Savaridas T , Serrano-Pedraza I , Khan SK , Martin K , Malviya A , Reed MR Reduced medium-term mortality following primary total hip and knee arthroplasty with an enhanced recovery program. Acta Orthop 2013;84:40–3. 23368747 10.3109/17453674.2013.771298 PMC3584601

[96] Gustafsson UO , Oppelstrup H , Thorell A , Nygren J , Ljungqvist O Adherence to the ERAS protocol is associated with 5-year survival after colorectal cancer surgery: a retrospective cohort study. World J Surg 2016;40:1741–7. 26913728 10.1007/s00268-016-3460-y

[97] Pisarska M , Torbicz G , Gajewska N , Rubinkiewicz M , Wierdak M , Major P , Compliance with the ERAS protocol and 3-year survival after laparoscopic surgery for non-metastatic colorectal cancer. World J Surg 2019;43:2552–60. 31286185 10.1007/s00268-019-05073-0

[98] Meara JG , M Leather AJ , Hagander L , Alkire BC , Alonso N , Ameh EA , Global Surgery 2030: evidence and solutions for achieving health, welfare, and economic development. Lancet 2015;386:569–624. 25924834 10.1016/S0140-6736(15)60160-X

[99] Price R , Makasa E , Hollands M World health assembly resolution WHA68.15: “Strengthening emergency and essential surgical care and anesthesia as a component of universal health coverage” – addressing the public health gaps arising from lack of safe, affordable and accessible surgical and anesthetic services. World J Surg 2015;39:2115–25. 26239773 10.1007/s00268-015-3153-y

[100] Peters AW , Pyda J , Menon G , Suzuki E , Meara JG The world bank group: Innovative financing for health and opportunities for global surgery. Surgery 2019;165:263–72. 30274731 10.1016/j.surg.2018.07.040

[101] McQueen K , Oodit R , Derbew M , Banguti P , Ljungqvist O Enhanced recovery after surgery for low- and middle-income countries. World J Surg 2018;42:950–2. 29383424 10.1007/s00268-018-4481-5

[102] Hagander L , Leather A A realized vision of access to safe, affordable surgical and anaesthesia care. Br J Surg 2019;106:e24–6. 30620073 10.1002/bjs.11068

