# Supplementary material for: Modern perioperative medicine – past, present, and future
Source: Innov Surg Sci. 2019 Dec 5;4(4):123–31. doi: 10.1515/iss-2019-0014 (PMC8059350; doi:10.1515/iss-2019-0014)
Supplement: Supplementary file 1 [file iss-04-20190014-s001.pdf]

## Reviewer Assessment

Harry F. Dean, Fiona Carter and Nader K. Francis*

# Modern perioperative medicine – past, present, and future

https://doi.org/10.1515/iss-2019-0014
Received August 15, 2019; accepted September 16, 2019

***Corresponding author: Nader K. Francis,** Department of General Surgery, Yeovil District Hospital, Higher Kingston, Yeovil BA21 4AT, UK; Enhanced Recovery after Surgery Society (UK) c.i.c., Yeovil BA20 2RH, UK; and School of Social and Community Medicine, University of Bristol, Canynge Hall, 39 Whatley Road, Bristol BS8 2PS, UK, Tel.: (01935) 384244, E-mail: nader.francis@ydh.nhs.uk. https://orcid.org/0000-0001-8498-9175

## Editor Comments to Original Submission

| | |
|---|---|
| **Recommendation Term:** | **Accept** |
| **Overall Reviewer Manuscript Rating:** | **95** |

This is an excellent review of the topic showing great knowledge of the past and future development of perioperative medicine!

Open Access. © 2019 Dean H.F., et al., published by De Gruyter. This work is licensed under the Creative Commons Attribution 4.0 Public License.
